# B-cell Lymphoblastic Lymphoma Presenting as a Sinonasal Mass: A Case Report

**DOI:** 10.7759/cureus.58132

**Published:** 2024-04-12

**Authors:** Brandon J Vilarello, Patricia T Jacobson, David A Gudis, Jonathan B Overdevest

**Affiliations:** 1 Vagelos College of Physicians and Surgeons, Columbia University, New York, USA; 2 Department of Otolaryngology-Head and Neck Surgery, Columbia University Irving Medical Center, New York, USA

**Keywords:** precursor b-cell lymphoblastic lymphoma, bone marrow biopsy, hematological malignancy, acute sinusitis, b-cell acute lymphoblastic leukemia, sino-nasal mass

## Abstract

B-cell lymphoblastic lymphoma (B-LBL) is an abnormal proliferation of lymphocyte precursor cells located primarily outside of the bone marrow and peripheral blood, typically in the mediastinum or other lymph nodes. It is often a disease of childhood that presents with lymphadenopathy, fatigue, pallor, bone pain, and weight loss with laboratory findings of anemia and thrombocytopenia. Initial presentations prompted by head and neck manifestations are exceedingly rare.

A five-year-old girl with no significant past medical history presented with right facial swelling and mild proptosis on ophthalmologic evaluation. She was referred to a tertiary care facility by her local otolaryngologist for further management after computed tomographic imaging revealed right maxillary sinus opacification and erosion of the anterior maxillary bone. Her symptoms were initially responsive to prednisone and amoxicillin-clavulanate, and only right unilateral nasal discharge persisted with a near-complete resolution of other sinonasal symptoms. Notably, laboratory values, including complete blood count, were within normal limits. Given concern for the etiology of the bony erosion, the patient presented for a second opinion, where imaging and biopsy resulted in flow cytometry findings consistent with B-ALL/LBL. After a bone marrow biopsy, the ultimate diagnosis was Murphy’s stage III B-cell lymphoblastic lymphoma.

Malignant neoplasms of the sinonasal region are rare in children, where primary sinonasal B-LBL is a unique occurrence. Clinical features of sinonasal B-LBL in the paranasal sinuses may masquerade as pathologies such as acute sinusitis, orbital cellulitis, and benign tumors or polyps that can lead to a confounding diagnosis. In this case presentation, an initial response to steroids and antibiotics should not provide false reassurance when other features and signs, such as maxillary bone erosion, may suggest the presence of malignancy.

## Introduction

Cancer among the pediatric population is a relatively uncommon diagnosis, and malignant neoplasms of the nasal cavity and sinuses usually occur in the adult population. Acute lymphoblastic leukemia (ALL) is the most common childhood malignancy and is responsible for about one-quarter of all childhood cancers. It represents less than 1% of adult cancer overall and only 0.4% of all cancers combined in the USA. The incidence of ALL is highest in Spain, among Hispanic patients in Los Angeles, and among Caucasians in certain Canadian provinces and New Zealand [1]. Lymphoblastic lymphoma (LBL) and ALL share many clinical and biological characteristics, but lymphoma indicates abnormal cells in the lymph nodes with less involvement of the bone marrow and peripheral blood [2]. Common presenting symptoms for children with acute lymphoblastic leukemia/lymphoma include lymphadenopathy, bleeding or bruising, pallor associated with hematologic abnormalities, including cytopenias, and bone pain [2,3]. Childhood non-Hodgkin’s lymphoma most commonly presents in the head and neck with asymptomatic cervical lymphadenopathy [4]. Additionally, extranodal localization in the head and neck may present as asymmetric tonsillar enlargement or swelling in the nasopharyngeal region and base of the tongue with enlargement of the lymphoid tissue of Waldeyer’s ring. Evidence of cancer in the paranasal sinuses, orbits, or maxilla is less common [4]. Atypical presentations can occur, with the literature containing various case reports documenting varied symptomatology [5].

LBL constitutes only a small percentage of non-Hodgkin’s lymphomas, and no definitive risk factors have been previously identified. The management of B-cell LBL (B-LBL) typically involves similar chemotherapeutic regimens as ALL, as these diseases have similar cytogenetic and histological underpinnings [2]. In this report, we discuss an atypical presentation of B-LBL masquerading as acute rhinosinusitis.

## Case presentation

**Figure 1:**
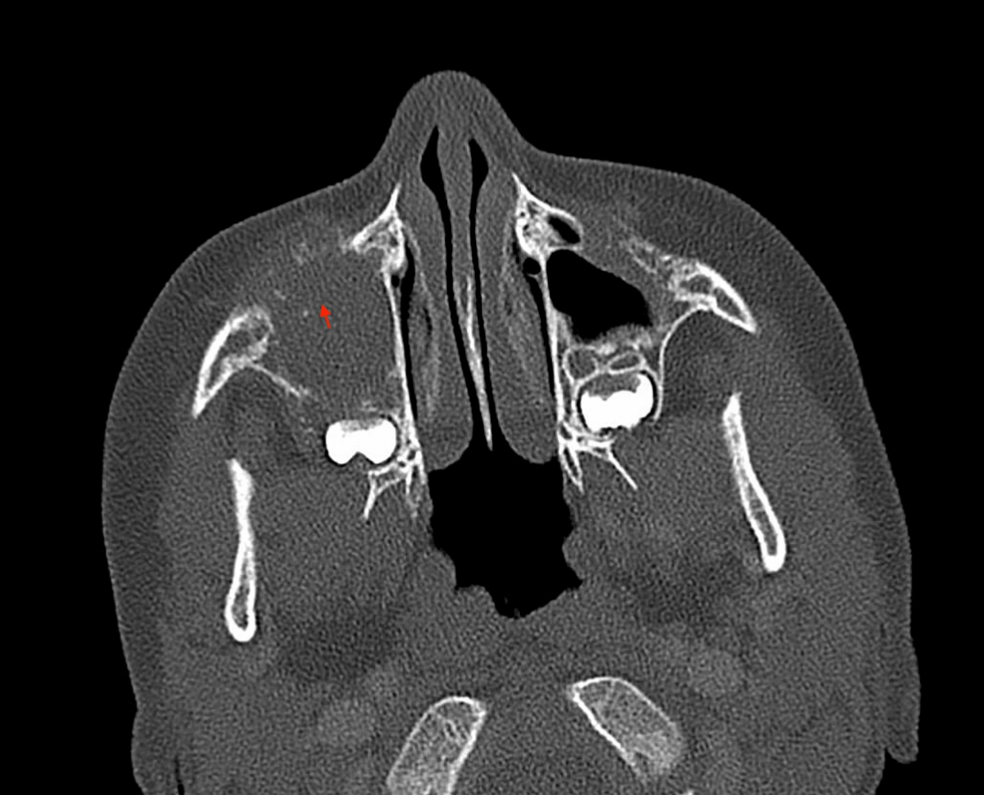
CT scan axial cut highlighting anterior maxillary bony erosion

**Figure 2:**
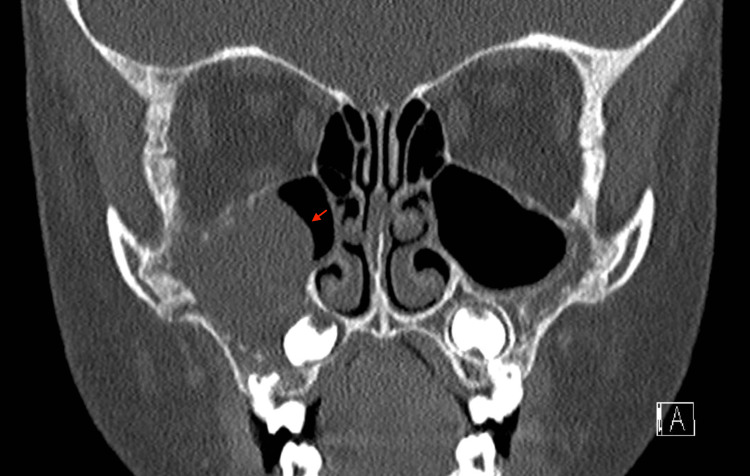
CT scan coronal cut showing near-complete right maxillary sinus opacification

**Figure 3:**
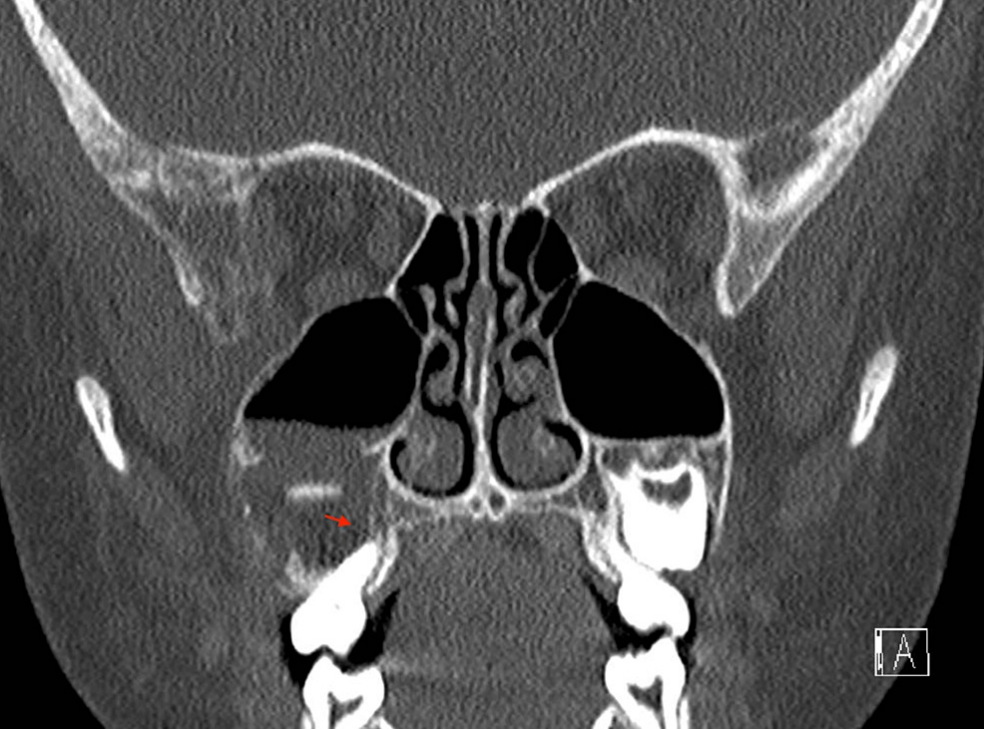
CT scan coronal cut showing partial bilateral maxillary sinus opacification and mimicking of periapical lucency (arrow)

A five-year-old girl with no significant past medical history presented with right facial swelling and mild proptosis on ophthalmologic evaluation. She was referred to a tertiary care facility by her local otolaryngologist for further management after computed tomographic (CT) imaging revealed right maxillary sinus opacification and erosion of the anterior maxillary bone, which is shown in Figures 1-3**.**

There was no documented organomegaly or lymphadenopathy on the physical exam. Her symptoms were initially responsive to prednisone and amoxicillin-clavulanate, and only the right unilateral nasal discharge persisted with a near-complete resolution of her facial swelling and ophthalmologic symptoms. Notably, laboratory values, including complete blood count, were within normal limits. Given concern for the etiology of the bony erosion, the patient presented for a second opinion, where imaging and a recommendation for biopsy resulted in flow cytometry findings consistent with B-ALL/LBL. After a bone marrow biopsy, the ultimate diagnosis was Murphy’s stage III B-LBL. Positron emission tomography (PET)-CT was significant for multiple bony lesions both above and below the diaphragm. The patient underwent induction, consolidation, and maintenance chemotherapy per the AALL1732 high-risk protocol [6]. At the submission of this article, the patient was in remission with no evidence of disease on the PET scan, and further treatment was transferred to a local hospital.

## Discussion

This case report highlights an atypical presentation of B-LBL with clinical symptoms mimicking acute rhinosinusitis. The patient’s symptoms initially appeared to respond to antibiotics and steroids, but the presence of bony erosion on the CT scan warranted further investigation. Astute clinical suspicion and pursuit of surgical biopsy ultimately led to appropriate clinical care and therapy for this patient.

Sinonasal non-Hodgkin’s lymphoma accounts for only 1.5% of all non-Hodgkin’s lymphomas in the United States [7]. Sinonasal lymphoma is rare in Western countries and often presents in the fourth or fifth decade of life in a 2:1 male-to-female ratio [8]. Common presenting signs and symptoms of sinonasal lymphoma include rhinorrhea, epistaxis, nasal obstruction, sinus infection, and proptosis [7]. Nasal obstruction has often been reported as the most frequent presenting symptom [9]. Other possible presenting symptoms include purulent nasal discharge, facial swelling, visual disturbance, and headache [9]. Though our patient had sinus symptoms and facial swelling, her clinical presentation was easily attributed to a more common, more benign cause. Because the symptoms often masquerade as other more common ailments, medical attention is often delayed [8]. Estimates of five-year survival are varied, ranging from 24% to 65%, and better prognosis is associated with earlier staging, smaller lesions, absence of B symptoms, normal lactate dehydrogenase, no lymphadenopathy, and T-cell lymphomas [8].

From our research and literature review, we were able to find very few cases of primary B-cell lymphoblastic leukemia/lymphoma presenting in the sinonasal region among pediatric patients. Belgaumi et al. presented two cases of pediatric patients with primary B-LBL of the paranasal sinuses. Both patients had palpable masses on their cheeks as well as supraclavicular lymphadenopathy [10]. Wang et al. also presented a case of a patient with primary B-cell lymphoblastic leukemia that initially presented as a progressively enlarging maxillary sinus mass without further symptoms [11]. Burton et al. published a case of a 19-year-old who had a relapse of ALL, which occurred in the ethmoid sinus and presented as facial pain and a supraorbital and paranasal mass [12]. These cases demonstrate similar findings to our case in that our patient experienced cheek swelling and had imaging that demonstrated a maxillary sinus mass.

Other than the difficulty associated with a rare diagnosis, the presenting signs and symptoms of a patient with sinonasal lymphoma can mimic various common pathologies, including a broad differential of many inflammatory, infectious, and neoplastic origins with the presence of both atypical and inflammatory lymphocytes on histology [8]. These diagnoses may include acute sinusitis, periorbital cellulitis, and malignant or non-malignant tumors [13]. What complicated our patient’s picture were the persistent sinonasal symptoms and the significantly improved facial swelling with prednisone and amoxicillin-clavulanate. The patient also had a CT scan after treatment, which showed an improved appearance of sinus disease, thus helping reassure providers that the opacified maxillary sinus was likely due to infection instead of a malignant neoplasm. Furthermore, there were no abnormalities in the patient’s CBC that would indicate a hematologic malignancy. An important imaging finding was the bony erosion of the right maxilla and the mildly persistent facial swelling that, though largely improved, did not completely resolve. With these findings, neoplasm could not be ruled out, and a biopsy was needed.

In a study conducted by Yen et al., sinonasal lymphoma was misdiagnosed as rhinitis/rhinosinusitis in 37.5% of patients due to overlapping symptoms and patient presentation [14]. Yen et al. determined that simple punch biopsies were not sufficient for making a diagnosis [14]. Fortunately, our patient underwent endoscopic sinus surgery and a maxillary antrostomy with biopsy, allowing for a pathologic diagnosis.

## Conclusions

Initial presentation of B-LBL as a sinonasal mass is rare and can easily be misdiagnosed as a non-malignant disease process. In this case, the characteristic clinical signs and symptoms of malignancy were absent, and the reported symptoms were easily attributed to more common disease presentations. Pediatric patients often fail to thoroughly and exhaustively describe their symptoms, so careful attention to clinical findings, like bony erosions, and high clinical suspicion are critical in ensuring proper management.
